# Composition of the Protein Ingredients from Insoluble Oat Byproducts Treated with Food-Grade Enzymes, Such as Amylase, Cellulose/Xylanase, and Protease

**DOI:** 10.3390/foods10112695

**Published:** 2021-11-04

**Authors:** Gilda Aiello, Yuchen Li, Ruoxian Xu, Giovanna Boschin, Grazina Juodeikiene, Anna Arnoldi

**Affiliations:** 1 Department of Human Science and Quality of Life Promotion, Telematic University San Raffaele, 00166 Rome, Italy; 2 Department of Pharmaceutical Sciences, University of Milan, 20133 Milan, Italy; yuchen.li@unimi.it (Y.L.); ruoxian.xu@unimi.it (R.X.); giovanna.boschin@unimi.it (G.B.); anna.arnoldi@unimi.it (A.A.); 3 Department of Food Science and Technology, Kaunas University of Technology, Radvilenu rd. 19, LT-50254 Kaunas, Lithuania; grazina.juodeikiene@ktu.lt

**Keywords:** circular economy, enzymatic treatment, oat press cake, plant proteins, plant-based beverages, phenols

## Abstract

The manufacture of plant-based drinks has the drawback of a huge production of underexploited press cakes. In particular, the oat press cake is mainly used in feed formulation, whereas added-value applications in human nutrition are scarce. Considering that enzymatic treatments may be useful to improve the nutritional quality of these insoluble byproducts, this study aimed to evaluate whether the treatment with some food-grade enzymes, such as amylase, cellulase/xylanase, protease, and their combination, may be useful to achieve this goal. Proteomic and peptidomic studies showed that the enzymatic treatments improved the protein extraction yields and induced a release of low molecular weight (LMW) peptides that were demonstrated to provide a useful antioxidant activity. In the treated oat press cake proteins, the concentration of the bound phenolic compounds was decreased, with the exception of caffeic acid, which was increased, and avenanthramides, which remained unchanged. Finally, the enzymatic treatment decreased the concentration of phytic acid. All these results indicate that the enzymatic treatments may be useful to ameliorate the nutritional profile of these protein ingredients, before their inclusion in different food products.

## 1. Introduction

A plant-based diet and plant proteins are becoming more and more important to meet the nutritional requirements of the growing human population, as well as to reduce the negative impact of food production on the environment. In this context, the valorization of sidestreams is certainly one of the main challenges for boosting the environmental and economic sustainability. Some food byproducts, such as those derived from the production of plant-based drinks, are currently among the most interesting waste materials owing to their high nutritional potential.

Compared with other cereals, oat (*Avena sativa*) can tolerate harsher growing conditions, such as a wet climate and acidic soil, and is, therefore, more resilient than other crops [1]. The health benefits of oat are attributed to its multifunctional characteristics and nutritional profile, it being an important source of nutrients and phytochemicals, i.e., well-balanced proteins, essential amino acids, fatty acids, dietary soluble fiber such as β-glucan, and phenolic compounds [1]. Among phenolics, a predominant position is occupied by avenanthramides (AVNs) and avenalumic acids, which are unique to oat among cereals. These compounds have been shown to possess an antioxidant activity in vitro and in vivo and are believed to be present also in the byproducts deriving from the production of oat beverages [2].

In view of making the agro-industry more sustainable and competitive by the valorization of the sidestreams, enzymatic treatments are nowadays widely employed. They aim to reduce the cell wall rigidity and to improve the extraction yields of protein and small molecules, such as phenols, and represent a promising method for improving the nutritional quality of these byproducts. The use of carbohydrate-degrading enzymes may improve the release of proteins from the matrix; in fact, plant proteins are partially linked to the lignocellulosic fraction, as reported in leaves, kernels, and other residues [3].

The enzyme-assisted treatment of citrus byproducts has already been proven to provide some advantages, such as a change in the phenolic profile and enhanced antioxidant activity [4,5,6], whereas the carbohydrase-assisted protein extraction has been applied to rapeseed press cake [7], oat bran [8], and defatted soybean flour [9]. The treatment of oat bran with commercial carbohydrases (i.e., Viscozyme, Celluclast, alpha-amylase, and amyloglucosidase) greatly increases the content of soluble phenolic compounds and the antioxidant activity [10]. Carbohydrases may, in fact, effectively hydrolyze the plant cell wall polysaccharide matrix and release valuable compounds, such as protein and phenols [11]. Typically, enzyme formulations include cellulases, hemicellulases, and pectinases [12]. However, whilst the technological processing facilitates the digestibility and bioavailability of nutrients by disrupting the food matrix, it may impair the technological and nutritional functionalities by altering the structure of its components (e.g., depolymerization of β-glucan and/or protein denaturation) and/or the interaction between them.

To the best of our knowledge, no research has evaluated the protein/peptide and phenol profile changes induced by the treatment of the solid byproducts from the production of oat-based drinks with food-grade enzymes, such as amylase, cellulase/xylanase, and proteases. This study, therefore, aimed to investigate the effects of multiple enzymatic treatments on the composition of protein concentrates and phenol extracts from an oat press cake. High-performance liquid chromatography coupled with mass spectrometry (HPLC–MS/MS) and data analysis were used to investigate the proteins, peptides, and phenolic profiles. Finally, the antioxidant activities of 3 kDa fractionated peptides were measured and the antinutritional factor phytic acid was quantified.

## 2. Materials and Methods

### 2.1. Materials and Reagents

All chemicals and reagents were of analytical grade and from commercial sources. Acetonitrile (ACN), Tris(hydroxymethyl)aminomethane (Tris–HCl), hydrochloric acid (HCl), ammonium bicarbonate, 2-iodoacetamide (IAM), 1,4-dithiothreitol (DTT), and trypsin from bovine pancreas (T1426, lyophilized powder, ≥10,000 units/mg protein) were from Sigma-Aldrich (St. Louis, MO, USA). Bovine serum albumin (BSA), Mini-Protean apparatus, precision plus protein standards, Bradford reagent, and Coomassie Blue G-250 were purchased from Bio-Rad (Hercules, CA, USA). The liquid bacterial α-amylase used for starch hydrolysis and the liquid preparation Cellustar XL (containing cellulase and xylanase) used for the hydrolysis of non-starch polysaccharides were obtained from AB Baltic Enzymes (Vilnius, Lithuania), whereas the neutral protease SQzyme PS-NL used for protein hydrolysis was from SUNTAQ (Guangzhou, China).

### 2.2. Enzymatic Treatments of the Oat Press Cake and Protein Extraction

The oat press cake (moisture content 64.17 ± 0.08%) from an industrial oat drink production was provided by a German company in a frozen state and stored at −18 °C. Its composition is shown in Table 1. After defrosting at room temperature, grinding by a laboratory grinder, and passage through a 200 µm sieve, the oat press cake meal was dissolved in distilled water in a 1:3 (*w*/*v*) ratio until the formation of a homogenous slurry, and then hydrolyzed with the different enzymes or enzyme combinations. The amounts of enzymes added to 100 g of slurry were the following: amylase 100 AU, cellulase/xylanase 400 AU, and protease 200 AU. The enzymatic activities are those indicated by the producing companies.

The characteristics of these enzymatic treatments are reported in Table 2. Each hydrolysis was performed at 50 °C for 90 min, then each suspension was centrifuged for 15 min at 3000× *g*. The supernatants were collected and the proteins were precipitated by adding 0.1 N hydrochloric acid until pH 5.0. The oat press cake proteins were collected by centrifugation and then freeze dried. The procedure for protein precipitation was performed also on the untreated oat press cake meal to obtain the control sample. The samples were thus the following: untreated oat press cake protein (Oat_Ctrl), protein from the oat press cake treated with amylase (Oat_Amy), protein from the oat press cake treated with cellulase/xylanase (Oat_Cxl), and protein from the oat press cake treated with amylase + cellulase/xylanase + protease (Oat_Mix).

### 2.3. Analysis by SDS–PAGE and Mass Spectrometry

Each protein sample was suspended in 10 mL of 100 mM Tris−HCl/0.5 M NaCl buffer at pH 8.0 at 4 °C overnight [13] and any solid residue was eliminated by centrifugation at 10,000× *g* for 30 min at 4 °C. The protein content of each solution was then assessed according to the Bradford method using BSA as standard for the calibration curve. The molecular weight distributions of the proteins from the untreated and treated samples were determined using reducing dodecyl sulfate–polyacrylamide gel electrophoresis (SDS–PAGE). The solutions were prepared by mixing 15 μL of each sample with 10 μL of Laemmli buffer (4% SDS, 20% glycerol, 10%, 0.004% bromophenol blue, and 0.125 M Tris−HCl, pH 6.8). Each solution was boiled for 5 min at 95 °C and then 25 μL were loaded in each lane of the gel, which was composed of a 4% polyacrylamide stacking gel over a 12% resolving polyacrylamide gel. The electrophoresis was conducted at 100 V until the dye front reached the gel bottom. Staining was performed with colloidal Coomassie Blue and destaining with 7% (*v*/*v*) acetic acid in water. The gel image was acquired by using the Bio-Rad GS800 densitometer and analyzed by using the software quantity One 1-D. Gel bands for all samples were sliced, digested with trypsin [14], and analyzed by nano-HPLC-CHIP-ESI Ion Trap using the experimental conditions previously reported [13]. The MS data were analyzed by Spectrum Mill Proteomics Workbench (Rev B.04.00, Agilent, Santa Clara, CA, USA), consulting the *A. sativa* (2508 entries) protein sequences database down-loaded from the National Center for Biotechnology Information (NCBI).

### 2.4. Circular Dichroism (CD) Spectroscopy

CD spectra were recorded in continuous scanning mode (190–300 nm) at 25 °C using a Jasco J-810 (Jasco Corp., Tokyo, Japan) spectropolarimeter. All spectra were collected using a 1 mm path-length quartz cell and averaged over three accumulations (speed 50 nm min^−1^). A reference spectrum of distilled water was recorded and subtracted from each spectrum. The estimation of the peptide secondary structure was achieved by using the method proposed in the literature [15,16].

### 2.5. Degree of Hydrolysis and Free Sulfhydryl Group Determination

The degree of hydrolysis (DH) of each sample was measured by the o-phthaldialdehyde (OPA) assay [17]. The sulfhydryl groups at the surface of the oat press cake proteins were determined according to a method proposed in the literature [18] with some modifications. Briefly, the Ellman’s reagent was prepared as follows: 4 mg of DTNB reagent was added to 1 mL of Tris–glycine buffer (0.086 M Tris, 0.09 M glycine, 4 mM EDTA, pH 7.0). Each solution was diluted in Tris–glycine buffer (*w*/*v* 0.15%). Then, 5 μL of Ellman’s reagent was added to 200 μL of protein suspension. The resulting protein suspensions were incubated at room temperature for 15 min under shaking and then centrifuged at 10,000× *g* for 10 min at room temperature. The absorbance was then recorded at 412 nm. A buffer solution without proteins was used as a reagent blank.

### 2.6. 3 kDa Fractionation, Peptide Content, and LC–MS Analysis

To collect the peptides released by the enzymatic treatment, the untreated and treated oat press cake protein samples (0.1 g) were solubilized in 1 mL of water. The solubilized peptides were fractionated by ultrafiltration, using membranes with a 3 kDa molecular weight cut-off (MWCO) (Millipore, Burlington, MA, USA). The peptide content was determined by o-phthalaldehyde (OPA) assay, following the procedure detailed in the literature [19] with some modifications. This assay is based on the formation of an adduct between the peptide α-amino group and OPA reagent by mixing 200 μL of OPA reagent with 20 μL of sample. After 1.5 min of incubation at 25 °C, the absorbance was measured at 340 nm using the Synergy H1 fluorescent plate reader (Biotek, Bad Friedrichshall, Germany). GSH (0–5 mg/mL) was used to build the calibration curve and the peptide content was obtained by interpolation. The peptides were analyzed by nano LC–MS/MS analysis according to chromatographic and MS condition reported in the Materials and Methods. Figure S1 shows the MSn TIC of the analyzed samples. The MS data were analyzed by Spectrum Mill Proteomics Workbench (Rev B.04.00, Agilent), consulting the *A. sativa* (22,508 entries) protein sequences database downloaded from the National Center for Biotechnology Information (NCBI). For MS/MS analysis and searching against a polypeptide sequence database, a non-enzyme-specific search considering all of the possible proteolytic cleavages was selected as a criterion. The percentage of amino acid composition was calculated by using the ExPASy-ProtParam tool by inserting as an input data set the sequences of the peptides identified by LC–MS.

### 2.7. Extraction of Bound Phenols

Briefly, 20 mg of untreated and treated oat press cake proteins were suspended in 400 μL of H_2_O and mixed thoroughly by vortex. The pH of solution was adjusted to 2 by adding 1 M HCl, then 10 μL of pepsin (4 mg/mL) was added and the solution was stirred for 1 h at 37 °C. This first digestion step was followed by a second one. The pH of solution was changed to 8 with 1 M NaOH, and 10 μL of trypsin (4 mg/mL), 10 μL of chymotrypsin (4 mg/mL), and 2 μL of pancreatin (4 mg/mL) were added. The digestion was performed at 37 °C for 2 h. After enzymatic digestion, ethanol (1.6 mL) was added to the mixture (to reach an 80% ethanol concentration), to extract the phenolic compounds. The solution was incubated under magnetic stirring overnight, centrifuged at 13,000× *g* for 5 min at room temperature, and the supernatant was collected. For the LC–MS injection, the supernatant was dried by Speed-Vac and then dissolved in 100 μL of 95% H_2_O, 5% ACN, 0.1% FA.

### 2.8. Phenolic Compound Identification and Quantification by MS

The quantification of gallic acid, vanillic acid, ferulic acid, caffeic acid, p-coumaric acid, cinnamic acid, and three AVNs (AVN A, AVN B, and AVN C) in the extracted phenolic fractions was performed by multiple reaction monitoring (MRM) mass spectrometry, monitoring one transition for each phenol. The monitored MRM transition for ferulic acid was *m*/*z* 585.3 → *m*/*z* 178.8, for p-coumaric *m*/*z* 165.2 → *m*/*z* 120.0, for caffeic acid *m*/*z* 181.2 → *m*/*z* 162.9, for gallic acid *m*/*z* 171.1 → *m*/*z* 142.9, for cinnamic acid *m*/*z* 149.3 → *m*/*z* 130.9, and for vanillic acid *m*/*z* 169.1 → *m*/*z* 142.9. The transitions monitored for the quantification of AVNs were: *m*/*z* 300.1 → *m*/*z* 147.0, *m*/*z* 330.1 → *m*/*z* 177.0, and *m*/*z* 316.1→ *m*/*z* 163.0 for AVN A, AVN B, and AVN C, respectively. The LC separation was performed applying the following gradient: 0% solvent B (0 min), 40% solvent B (0–10 min), 95% solvent B (10–20 min), and back to 0% in 15 min. The drying gas temperature was set at 300 °C, flow rate 3 L/min (nitrogen). Data acquisition was carried out in positive ionization mode. Capillary voltage was −1970 V, with endplate offset −500 V. Full-scan mass spectra were acquired in the mass range from m/z 50 to 600 Da. Three technical replicates (LC–MS/MS runs) were run for each sample. Analytical parameters, i.e., LOQ and LOD, were measured to ensure the appropriate performance of the developed method. The accuracy of the assay was assessed by spiking the untreated oat press cake protein sample with 25 µg/mL of each standard phenolic acid and 20 ppb of each AVN. The sensitivity of the method was calculated by the LOQ (signal-to-noise (S/N) = 10) and LOD (S/N = 3). The analytical validation study evaluated the assay accuracy, the intra-day precision linearity, and the recovery.

### 2.9. Radical Scavenging Activity of the 3 kDa Peptide Extracts measured by Ferric Reducing Ability (FRAP) Assay

The FRAP assay was carried out as described by Benzie and Strain [20], with minor modifications for working on a 96-well microplate. The FRAP reagent was prepared by mixing 25 mL of 300 mmol/L sodium acetate buffer, 2.5 mL of 10 mmol/L TPTZ solution, and 2.5 mL of 20 mmol/L FeCl_3_ solution in a 10:1:1 ratio. Each sample (20 mL) was mixed with 200 μL of FRAP reagent, mixed vigorously, and incubated at 37 °C for 10 min. The ferric tripyridyltriazine (Fe^III^-TPTZ) complex is reduced to ferrous tripyridyltriazine (Fe^II^-TPTZ) form in the presence of antioxidants and develops an intense blue color, with maximum absorption at 593 nm. Concentrations of 0 to 1000 μM FeSO_4_•7H_2_O were used for the calibration curve. The results are expressed as μmol/L of Fe^2+^ equivalents.

### 2.10. Phytic Acid (PA) Quantification

The untreated and treated oat press cake protein samples were lyophilized before phytic acid determination, which was performed following a colorimetric method [21]. Aqueous phytic acid solutions at the concentrations of 0–100 μg/mL were used for the quantification. Samples (100 µL) and standard solutions were diluted 25 times with 2.4 mL of ddH_2_O; then, 600 µL of the diluted samples and standards were combined with 200 µL of modified Wade reagent (0.03% of FeCl_3_ 6H_2_O and 0.3% of sulfosalicylic acid), and the absorbance was measured at 500 nm.

### 2.11. Total Carbohydrate Quantification

The total carbohydrate content of each sample was measured by the phenol sulfuric acid assay [22]. Untreated and treated oat press cake proteins (10 mg) were suspended in 1 mL of 1 M HCl, and then the solution was heated at 100 °C for 2 h. The solution was separated by centrifugation at 5000× *g* for 10 min at room temperature, and the supernatant was collected. An aliquot of 10 μL of supernatant was mixed with 100 μL of 5% (*w*/*w*) phenol solution and then 500 μL of concentrated sulfuric acid. The mixture was shaken for 25 min at 28 °C and the absorbance was measured at 490 nm.

### 2.12. Statistical Analysis

All experiments were performed in triplicate, and the data are presented as the mean ± standard deviation. The collected data were subjected to analysis of variance (ANOVA). Duncan’s multiple range test was used to analyze differences between treatments.

## 3. Results and Discussion

### 3.1. Effects of the Enzymatic Treatments on Bound Carbohydrates

**Figure 1:**
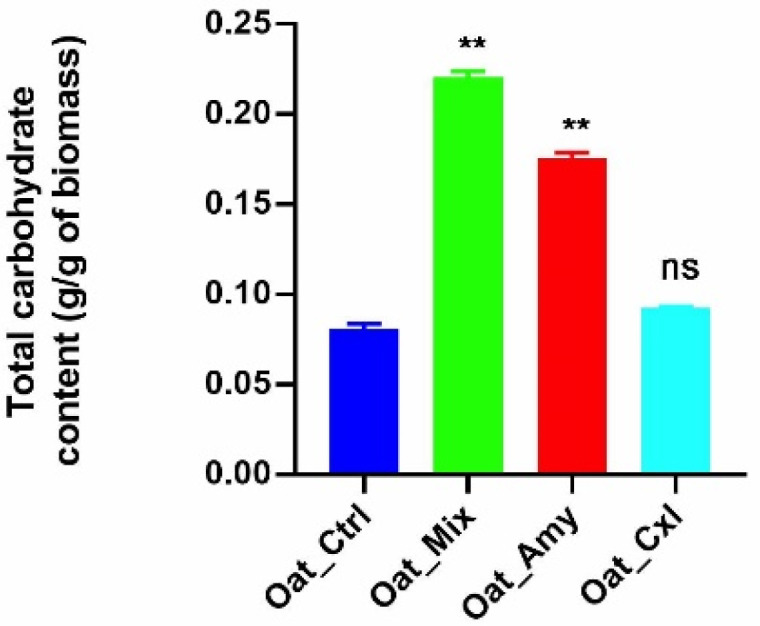
Total carbohydrate content determination. Statistical analysis was performed by one-way ANOVA. The data are presented as the means ± s.d. of three independent experiments, ** *p* < 0.001.

To investigate how the total carbohydrates of the oat press cake proteins had been affected by the enzymatic treatments, the phenol sulfuric acid assay was employed. Figure 1 shows that the enzymatic treatments induced the release of bound carbohydrates. In detail, increases of 54%, 12%, and 63% in the carbohydrate content were observed in Oat_Amy, Oat_Cxl, and Oat_Mix, respectively, versus Oat_Ctrl. It is not surprising that amylase was more efficient than the other enzymes in releasing bound carbohydrates, since starch is very abundant in the oat press cake (Table 1). However, the combination of cellulases and xylanase produced a moderate release of bound carbohydrates (with a low statistical significance) by a partial hydrolysis of the oat fiber polysaccharides.

### 3.2. Effects of the Enzymatic Treatments on the Primary Structure of the Oat Press Cake Proteins

**Figure 2:**
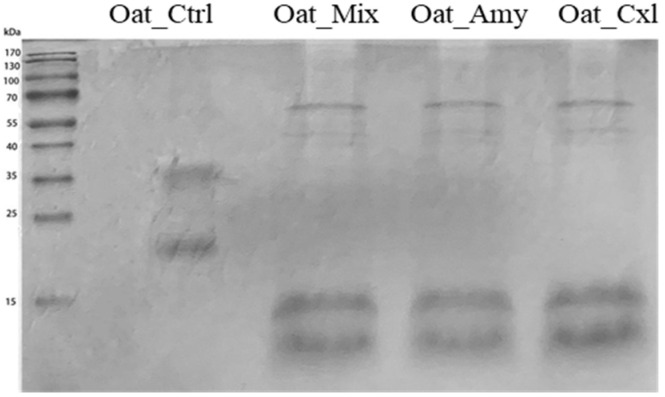
Reduced SDS–PAGE protein profile of untreated and enzymatically treated oat press cake proteins: M, pre-stained molecular marker; Oat_Ctrl, Oat_Amy, Oat_Mix, Oat_Cxl. Each sample (20 μL) was added to 10 μL of loading buffer, loading 30 μL for each well.

Proteins are largely responsible for the main characteristics of most foods, since their composition influences the nutritional, rheological, and sensory properties. Enzymatic treatments may induce chemical and structural modifications, impacting the nutritional features of the final products. The effects of the enzymatic treatments on the oat press cake proteins were initially explored by evaluating their molecular weight profile using electrophoresis in reducing conditions. Figure 2 shows the SDS–PAGE of the untreated and treated oat press cake protein samples. Gel bands were then sliced, digested with trypsin, and analyzed by nano-HPLC–ESI–MS/MS. The proteins identified in each sample are listed in Table 3.

The SDS–PAGE of Oat_Ctrl (Figure 2) shows two very intense bands, at 32–35 kDa and 22–24 kDa, that may be attributed to the 12S α-polypeptide and the 12S β-polypeptide, respectively. This profile is in line with the protein extract from the seed [23]. The protein profiles of treated oat press cake protein samples are completely different, suggesting that the enzymes had induced cleavage of the peptide bonds, i.e., partial hydrolysis of the proteins, indicated by the loss of intensity in the bands at 32–35 kDa and 22–24 kDa and the appearance of two intense bands at 10 kDa and 17 kDa. The nano-HPLC–ESI–MS/MS analysis (Table 3) indicated that these bands correspond to Avenins, Avena amylase trypsin inhibitors, Vromindolines, and Tryptophanin. Specifically, Vromindolines are starch-bound proteins that can contribute up to a 50% reduction in the oat grain hardness [24], whereas tryptophanins also contribute to oat grain softness, because they are bound to lipids [25]. It is thus possible to affirm that the enzymatic treatments efficiently disrupted the flour matrix to release these proteins, which were undetected in Oat_Ctrl, favoring in the meanwhile the degradation of the 11S and 12S storage proteins, as confirmed by the total spectrum intensity, which showed higher values in Oat_Ctrl compared to those detected in Oat_Amy, Oat_Cxl, and Oat_Mix. Interestingly, it was possible to identify hydroxyanthranilate-hydroxy-cinnamoyltransferase, which plays a pivotal role in the biosynthesis of the avenanthramides.

The proteolytic activity of all enzymatic treatments was confirmed by the degree of hydrolysis, which in Oat_Amy, Oat_Cxl, and Oat_Mix was higher by 86.5%, 84.5%, and 87.4%, respectively, than in Oat_Ctrl.

### 3.3. Effects of the Enzymatic Treatments on the Secondary and Tertiary Structure of the Proteins

**Figure 3:**
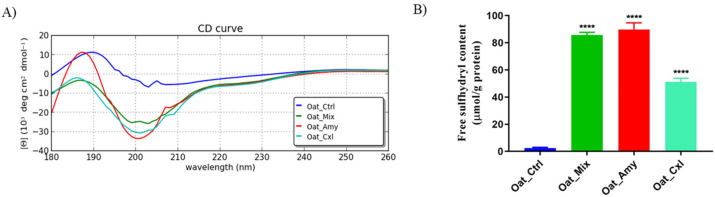
(**A**) CD spectra and (**B**) free SH group determination of Oat_Ctrl, Oat_Amy, Oat_Cxl, and Oat_Mix. The statistical analysis was performed by one-way ANOVA; **** *p* < 0.0001.

To investigate how the secondary and tertiary structure of the proteins had been affected by the enzymatic treatments, circular dichroism spectroscopy was employed and the content of free SH groups was measured. The CD spectra in the far UV region (190–230 nm) are shown in Figure 3A. One positive Cotton effect at 190 nm was observed for Oat_Ctrl, suggesting an α-helix-rich conformation. The enzymatic treatments induced a secondary negative Cotton effect with a minimum peak at 200 nm, indicating the predominance of β-sheet structures and random coils.

To obtain further information about the secondary structure of the oat press cake proteins, the BestSel tool was applied [26]. The results (Table 4) suggest that a reduction in the percentage of α-helices and an increase in β-sheet had been induced by the enzymatic treatment versus the untreated sample (Oat_Ctrl). Reductions in the α-helices up to 8.9%, 3.7%, and 5.9% for Oat_Mix, Oat_Amy, and Oat_Cxl, respectively, were observed versus the Oat_Ctrl (22.2%). In addition, increases in the β-sheet up to 20.3%, 28.0%, and 28.1% for Oat_Mix, Oat_Amy, and Oat_Cxl, respectively, were detected versus Oat_Ctrl (8.5%). In the meanwhile, parallel increases in random coils were observed.

The measurement of the content of free SH groups located on the protein surface was used to provide further insights into changes in protein tertiary structure caused by the enzymatic treatments. Figure 3B shows a significant increase in free SH groups after the enzymatic treatments. In detail, the free SH contents of Oat_Mix, Oat_Amy, and Oat_Cxl were 85.7 ± 2.0, 89.9 ± 4.9, and 51.09 ± 2.8 μmol/g, respectively. All these values are much larger than the value of untreated oat press cake protein (Oat_Ctrl), equal to 2.5 ± 0.6 μmol/g (*p* < 0.00015). The SH content increase might be mainly attributed to the conversion of disulfide bonds into sulfhydryl groups, leading to protein unfolding and dissociation [27]. The proteolysis has been reported to be involved in the increment in the SH group by releasing free amino acids and other water-soluble components [28]. The alteration of the structure could reflect the change in the protein internal hydrogen bonds, hydrophobic bonds, and tightness of intermolecular binding [29]. These structural changes could expose more functional groups inside the protein molecule, thereby changing the antioxidant activity and the functional properties of the proteins. In addition, phenolic compounds, such as chlorogenic acid, avenanthramides, quercetin, and gentianic acid, could preferentially react with free radicals, leading to a protective effect on the SH groups [30].

### 3.4. Analysis of the 3 kDa Peptide Fractions by Liquid Chromatography–Mass Spectrometry (LC–MS)

Since LMW peptides (cut-off 3 kDa) are known to provide useful health benefits [13,31,32], it was decided to evaluate whether the enzymatic treatments influenced their formation. The quantification of the peptides, performed by the OPA assay, indicated that Oat_Mix contained the highest concentration of peptides (0.49 ± 0.02 mg/mL), whereas the concentrations in Oat_Amy and Oat_Cxl were 0.45 ± 0.01 mg/mL and 0.44 ± 0.02 mg/mL, respectively. All these values are much higher than the value of Oat_Ctrl (0.010 ± 0.001 mg/mL). The enzymatic treatment therefore highly incremented the concentration of these potentially bioactive compounds.

The samples were then submitted to LC–MS analysis, whose results are reported in Table S1 in the Supplementary Materials (complete list) and in Table 5 (only peptides belonging to storage proteins). In agreement with the OPA results, the total number of identified peptides were 160 in Oat_Mix, 124 in Oat_Amy, 36 in Oat_Cxl, and only 11 in Oat_Ctrl (Table S1 in the Supplementary Materials). Focusing the attention only on the storage proteins, which are the most abundant proteins in oat seeds (Table 5), it is not surprising that the treatment with the enzyme combination including a protease (Oat_Mix) released nine peptides belonging to the 11S and 12S globulins, Avenacosidase, Vromindoline, and Gliadin-like avenin, whereas the treatment with amylase (Oat_Amy) released six peptides belonging to 11S globulin, avenin, Gliadin-like avenin, and Avenacosidase 1, and the treatment with cellulase/xylanase released only one peptide belonging to Gliadin-like avenin. Only one peptide belonging to a storage protein was identified in the control sample.

### 3.5. Evaluation of the Antioxidant Activity of the Oat Press Cake Proteins and Low Molecular Weight Peptides

It was decided to assess the potential antioxidant activity of the oat press cake proteins using the FRAP assay, which showed that all of the samples were endowed with an antioxidant activity that was, however, higher in the enzymatically treated samples. Specifically, FRAP levels were significantly increased by 40.2% for Oat_Mix (220.7 μmol/L of Fe^2+^ equivalents), 41.1% for Oat_Amy (224.07 μmol/L of Fe^2+^ equivalents), and 33.6% for Oat_Cxl (198.87 μmol/L of Fe^2+^ equivalents), respectively, versus Oat_Ctrl (131.9 μmol/L of Fe^2+^ equivalents).

**Figure 4:**
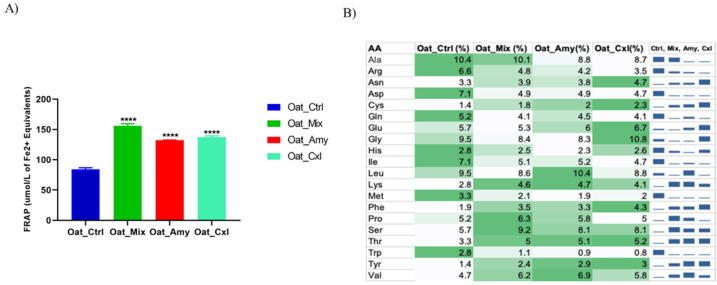
Antioxidant activity of the low molecular weight peptide fractions. (**A**) Results of the FRAP assay. Bars represent the average ± SD of 3 independent experiments in duplicate. **** *p* < 0.0001 versus untreated sample. (**B**) Amino acid (AA) compositions of the samples.

The fact that oligopeptides derived from hydrolyzed oat proteins have been reported to have biological activities, including antioxidant properties [33], encouraged us to investigate the contribution of 3 kDa peptides to the total antioxidant activity. The results are shown in Figure 4A. The treatments increased the FRAP value by 85%, 57%, and 64%, for Oat_Mix, Oat_Amy, and Oat_Cxl, respectively, compared to Oat_Ctrl (*p* < 0.0001), highlighting a significant enhancement of the antioxidant activity of the released peptides.

The literature indicates that antioxidant peptides are characterized by hydrophobic amino acids, such as Leu or Val, in their N-terminal regions, aromatic amino acid residues (Phe, Trp, Tyr, and His), and nucleophilic sulfur-containing amino acid residues (Cys and Met) [34,35]. Another amino acid that may also contribute to the antioxidant activity is Lys [36]. In addition, amino acids with aromatic side chains can also donate protons to the electron-deficient radicals, further improving the radical scavenging property. Figure 4B compares the amino acid compositions of the analyzed samples; indeed, some specific amino acids (such as Val, Phe, Tyr, Ser, Thr, and Lys) are more represented in the treated oat press cake LMW peptides. For example, the content of Phe is 2.2-fold higher and that of Tyr 2-fold higher in Oat_Cxl peptides than in the Oat_Ctrl ones, and the content of valine in Oat_Amy peptides is 1.5-fold higher than in Oat_Ctrl ones. According to previous studies, some di- and tripeptides with aromatic amino acid residues (Tyr or Trp) and valine are highly likely to have strong antioxidant activity [37]. Particularly, the peptides LVYIL and YHNAPGLVYIL have been reported to be associated with increased activities of antioxidant enzymes, with a 29% increase in cell viability [33].

### 3.6. Identification and Quantitation of the Main Phenolic Compounds by HPLC–ESI–MS/MS

It is well documented that phenolics are concentrated in the outer seed coat/pericarp of the grain, that these compounds are often bound within the walls of plant cells, and that the antioxidant activity of the bran is higher than that of the refined endosperm [38]. It is also known that phenolic acids mostly occur as compounds bound to proteins, causing very low bioavailability [39], and that these interactions take place either via non-covalent (hydrophobic, ionic, and hydrogen bonds) or covalent bonds [40]. These facts suggest that the oat press cake may represent a potential source of these important phytochemicals, and it seemed feasible that the enzymatic treatments may improve the bioavailability of these phytochemicals.

After having verified that the phenols could not be directly extracted from the samples (data not shown), the analysis of the bound species was performed by HPLC–MS/MS after two sequential digestion steps with pepsin and trypsin. The chromatograms and MS/MS spectra are shown in Figure S1. It was possible to identify and quantify six phenolic acids (gallic acid, vanillic acid, ferulic acid, caffeic acid, p-coumaric acid, and cinnamic acid), and three AVNs (AVN A, AVN B, and AVN C). The identification was performed by comparing the retention times and the fragmentation ions with those of authentic standards, whereas the quantification was based on standard curves, which showed high correlation values (R^2^~0.998).

Table 6 reports the concentrations of phenolic acids and the content of AVNsin the oat press cake protein samples.The quantitative analysis revealed that the oat press cake is an interesting added-value material, since Oat_Ctrl contains a much higher amount of vanillic acid (407.56 ± 60.73 μg/g DW) than eight cultivars of husked oat, among which the richest was the Peppi cultivar (7.05 ± 0.37 μg/g DW). In addition, the contents of p-coumaric acid, vanillic acid, gallic acid, and caffeic acid are also much higher (63.06, 407.56, 2894.26, and 4.72 μg/g, respectively) than those reported in oat bran (12 ± 0.22 μg/g of p-coumaric acid, 24 ± 2.4 μg/g of vanillic acid, 5.4 ± 0.15 μg/g of caffeic acid) as well as in oat grain (113.3 ± 2.6 μg/g of gallic acid) [41,42]. Instead, the amount of cinnamic acid (12.07 ± 0.38 μg/g) is comparable to that quantified in a Finnish husked oat [43], and ferulic acid is much lower (1.98 ± 0.07 μg/g) than the value detected in the Akseli variety (829 ± 73.8 μg/g) [43]. A comparison of the main phenolic acid profiles in Oat_Ctrl with those of some commercial oat products shows that the amount of p-coumaric acid is comparable, whereas the amount of vanillic acid is much higher [44]. In different oat cultivars, bound phenolic acids represent 89.6–97.3% of the total phenolic compounds and p-coumaric acid is mostly present in the bound fraction, accounting for 59% of total bound compounds [15]. Similarly, in different oat products, bound phenolics were from two to ten times more concentrated than the free species, with vanillic acid, caffeic acid, and p-coumaric acid mostly present in the bound form [44].

There are, however, significant changes in the phenolic compound profiles induced by the enzymatic treatments. The results in Table 6 clearly indicate that most phenolic acids, in particular cinnamic acid, p-coumaric acid, vanillic acid, gallic acid, and ferulic acid, decrease in the treated oat press cake protein versus Oat_Ctrl. The effects are more evident in Oat_Mix and Oat_Cxl. The fact that enzymes such as cellulase and xylanase greatly influence the polyphenol content underlines the role of cellulose and xylans in the binding of these phytochemicals to proteins. On the contrary, the only phenolic acid that was increased was caffeic acid, again, especially in Oat_Mix and Oat_Cxl. Another paper observed a significant increase in caffeic acid after treatment with cellulase [45].

AVNs are a group of unique phenolic acid derivatives typical of oats. The Oat_Ctrl sample contains an amount of AVNs lower than that reported in the literature for oat grain (40–130 µg/g) [46]. However, the various levels of AVNs analyzed in oat varieties from Finland and China depend on the cultivar, geographic location, environment, and genetics, which play crucial roles in the generation of secondary metabolites such as AVNs [46]. The total amount of these phytochemicals is smaller in the enzymatically treated samples. This depends mostly on the relevant decrease in ANV B, whereas the contents of AVN A are slightly higher in Oat_Amy, Oat_Cxl, and Oat_Mix, as well as those of AVN C in Oat_Mix and Oat_Cxl. While most phenolic compounds are often covalently bound to proteins, AVNs belong to the free phenolic acid fraction [44], a fact that can explain why their residues are very small in the byproducts and in these samples [15].

### 3.7. Effects of Enzymatic Treatments on the Phytic Acid Content

**Figure 5:**
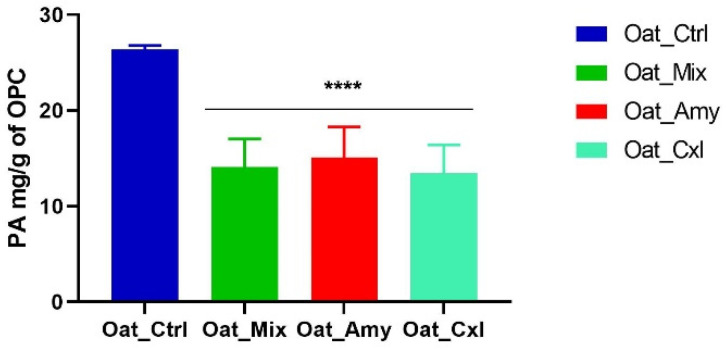
PA content determination. Statistical analysis was performed by one-way ANOVA (****) *p* < 0.0001. The data are represented as the means ± s.d. of three independent experiments.

In order to check the potential release of anti-nutritional factors induced by the enzymatic pre-treatment, phytic acid was quantified. Figure 5 shows that the enzymatic treatments and protein precipitation reduced the phytic acid. In detail, a reduction in the PA content by 46 ± 1%, 42 ± 1%, and 46 ± 1% was observed in Oat_Mix (14.08 ± 0.02 mg/g), Oat_Amy (15.11 ± 0.02 mg/g), and Oat_Cxl (13.43 ± 0.05 mg/g), respectively, versus Oat_Ctrl (26.38 ± 0.5 mg/g). This may be explained by considering that phytate is mainly present in the form of water-soluble salts, such as sodium and potassium phytate. By improving the protein solubility, the enzymatic treatment by cellulase, xylanase, and proteases increased the passive diffusion of water-soluble phytates during the exposure to water [47]. Successful applications of enzymes for treating raw material have been already described as efficient in reducing the phytic acid content [48].

## 4. Conclusions

Here, we provide evidence that the treatment of the oat press cake, a byproduct which is generally discarded after oat drink preparation, with enzymes, such as amylase, cellulase/xylanase, and protease, may improve its nutritional value. This is particularly relevant considering the increasing market for these kinds of products that are more and more appreciated by vegans, vegetarians, and flexitarians. The protein extraction was greatly facilitated by the enzymatic treatments, as well as the release of LMW peptides, with useful technological and nutritional consequences, such as improved water solubility. In addition, it is well known that LMW peptides provide useful health benefits, mainly in the area of metabolic syndrome prevention [12,32]. Here, in particular, we evaluated the antioxidant activity of these peptides, which indeed was superior after enzymatic treatment. These results underline the importance of peptides in the global antioxidant activity of these food ingredients; in fact, the higher antioxidant activity of treated oat press cake proteins cannot be explained by the phenols, whose concentrations are decreased by the enzymatic treatments, but only by the presence of antioxidant peptides. Finally, it seems possible to affirm that the application of amylase, cellulase/xylanase, and proteases (alone or in combination) represents a new strategy to recover nutritional ingredients otherwise inaccessible from industrial byproducts. The recovery and reuse of these materials may obtain economic benefit by reducing the waste of natural sources.

## Figures and Tables

**Table 1:** Proximate analysis of the untreated oat press cake (%, d. m.).

Component	Percentage
Moisture	64.17 ± 0.08
Protein	32.42 ± 0.45 *
Lipids	7.79 ± 0.03 *
Insoluble dietary fibre	22.97 ± 0.15 *
Soluble dietary fibre	3.19 ± 0.02 *
Starch	27.32 ± 0.20 *

* Percentage of dry matter; data are expressed as mean value (*n* = 3) ± SD; SD—standard deviation.

**Table 2:** Characteristics of used enzymes.

Enzyme	Activity, U/g	Organism of Origin	Optimal pH	Optimal Temperature, °C
α-Amylase	>1400	*Bacillus licheniformis*	5.5–7.5	70–85
Cellulase/xylanase mixture	>45,000 cellulase >34,000 xylanase	*Trichoderma reesei*	5.0–6.5	40–60
Protease	116,350	*Aspergillus oryzae*	6.0–7.5	30–50

**Table 3:** Proteins identified in each sample, i.e., Oat_Ctrl, Oat_Amy, Oat_Mix, and Oat_Cxl, with their main MS/MS features.

Distinct Summed MS/MS Search Score	% AA Coverage	Total Spectrum Intensity	Protein MW	Protein pI	Accession (#)^(*a*)^	Protein Name
Oat_Ctrl						
27.86	4.8	6.61 × 108	58,999.9	9.22	P12615	12S seed storage globulin 1
27.86	7.9	6.61 × 10^8^	35,779.4	10.2	Q38781	Oat storage protein 12S globulin (Fragment)
18.96	2.3	6.55 × 10^8^	58,566.4	8.8	O49258	12S globulin
18.96	5.6	6.55 × 10^8^	24,685.5	7.94	P27919	Avenin
28.39	4.7	1.27 × 10^8^	59,804	9.52	Q38780	11S globulin
5.73	4.1	1.19 × 10^8^	59,773.6	5.38	A0A4Y5UJ50	4-coumarate:CoA ligase
6.26	9.4	6.95 × 10^8^	23,329.1	11.17	A0A3G1AXD2	Ribosomal protein S4
7.26	4.2	6.11 × 10^7^	53,845.5	5.16	A0A3G1AWG0	ATP synthase subunit beta
5.58	8.2	2.79 × 10^7^	28,805.3	5.98	I4IY74	Pollen allergen Ave s 5 (Isoallergen A)
6.65	1.4	2.17 × 10^7^	1,221,607	9.18	A0A3G1AUJ8	DNA-directed RNA polymerase subunit beta
5.77	17.7	1.55 × 10^7^	10,773.6	11.07	A0A3G1AU31	30S ribosomal protein S15
6.05	4.3	1.30 × 10^7^	50,203.8	8.69	Q941N4	Receptor kinase
**Oat_Mix, Amy, Cxl**						
5.55	14.6	1.15 × 10^9^	16,544	8.3	A0A1B2LQF1	Avena alpha amylase trypsin inhibitor
10.1	5.5	1.05 × 10^9^	78,923	9.03	A0A3G1ATL7	DNA-directed RNA polymerase subunit gamma
9.8	12.2	9.76 × 10^8^	30,425	5.28	I4IY75	Pollen allergen Ave s 5 (Isoallergen B)
15.5	4.2	6.07 × 10^8^	100,456	7.61	A0A482JYP4	Phototropin-like protein
11.78	6.1	5.38 × 10^8^	59,614	5.39	P54411	T-complex protein 1 subunit epsilon
4.57	3.9	4.67 × 10^8^	58,836	8.14	F5B4I6	Non-specific serine/threonine protein kinase
6.22	7.6	2.74 × 10^8^	32,980	8.86	Q7XXP0	Hydroxyanthranilate hydroxycinnamoyltransferase 4 (Fragm)
5.31	1.8	1.75 × 10^8^	96,836	5.66	G1JSL5	Lipoxygenase
15.08	16.6	1.66 × 10^8^	41260	6.6	D5L0B2	Putative 2-oxoglutarate dependent dioxygenase
21.65	5.2	1.59 × 10^8^	170,146	7.26	A0A3G1AXC3	RNA polymerase beta subunit
12.49	8.6	1.31 × 10^8^	53,847	5.16	A0A3G1AWG0	ATP synthase subunit beta
15.26	4.2	1.26 × 10^8^	126,405	5.75	P06594	Phytochrome A type 4
11.29	9.6	1.14 × 10^8^	49,110	5.66	A0A481SVJ0	Phenylalanine ammonia lyase II (Fragm)
5.82	11.9	1.10 × 10^8^	16,508	8.71	R4I3I8	Vromindoline 3
5.82	11.9	1.10 × 10^8^	16,482	8.34	A7U440	Tryptophanin
13.9	12.5	9.08 × 10^7^	53,822	6.17	Q43380	Non-specific serine/threonine protein kinase
7.36	5.9	8.75 × 10^7^	67,169	6.06	P22220	Arginine decarboxylase
5.33	6.3	8.38 × 10^7^	35,780	10.13	Q38781	Oat storage protein 12S globulin (Fragm)
13.55	14.9	7.25 × 10^7^	22,640	5.5	A0A2P0ZEN0	Ribulose bisphosphate carboxylase large chain (Fragm)
13.08	5.9	3.89 × 10^7^	112,385	6.48	Q38766	Glycine cleavage system P protein
6.91	10.8	3.73 × 10^7^	20,706	6.13	A0A0R6HRG0	Ribulose bisphosphate carboxylase large chain (Fragm)
5.29	3.8	2.01 × 10^7^	42,153	9.01	Q9LLD7	Fructose-bisphosphate aldolase
6.83	3.4	1.82 × 10^7^	65,678	6.03	Q38786	Avenacosidase 1
3.71	1.2	1.69 × 10^7^	105,789	8.63	A0A3Q8R3E1	Cellulose synthase-like CslF6
3.68	13.9	1.39 × 10^7^	18,766	7.84	Q071L4	Aluminum-activated malate transporter (Fragm)
3.46	20.9	1.05 × 10^7^	9356	8.8	A0A2L0U0E7	Defensin 16
7.28	4.6	4.29 × 10^6^	53,776	9.36	O49257	12S globulin
7.28	4.1	4.69 × 10^6^	59,811	9.14	Q38780	11S globulin

(*a*) #: Accession number reported in UniprotKB. Protein identified as belonging to *A. sativa*.

**Table 4:** Percentage of secondary structure composition of the oat press cake extracted proteins.

	α-Helix (%)	β-Sheet (%)	Turn (%)	Others (%)
Oat_Ctrl	22.2	8.5	32.7	36.6
Oat_Mix	8.9	20.3	13.7	57.1
Oat_Amy	3.7	28.0	7.2	61.2
Oat_Cxl	5.9	28.1	7.4	58.6

**Table 5:** Peptides from storage proteins identified in 3 kDa fractions of Oat_Ctrl, Oat_Amy, Oat_Mix, and Oat_Cxl.

Spectrum Intensity	Peptide Sequence	*m*/*z* (Da)	% AA Coverage	MH^+^ (Da)	Peptide pI	Protein MW (Da)	Accession #	Protein Name
Mix								
1.40 × 10^8^	SQQGPVEHQAYQPIQS	599.57	1.2	1796.9	5.22	58,674.5	P14812	12S seed storage globulin 2
4.13 × 10^7^	ALGISQQAAQRIQSQNDQRGEI	804.27	1.3	2411.2	6.12	59,404.6	Q38780	11S globulin
4.09 × 10^7^	DLGADVR	746.06	3.0	745.4	4.21	65,692.1	Q9ZP27	Avenacosidase 2
3.48 × 10^7^	YQPIQSQEGQSTQYQVGQSTQ	795.80	3.1	2385.1	4	58,224.1	O49258	12Sglobulin
1.44 × 10^7^	QQSEIMKQVHVAQTLPSK	684.75	2.5	2052.1	8.6	15,927.3	R4I3I8	Vromindoline 3
5.96 × 10^6^	TNPNSMVSHIAGKSSILRALPVDVLAN	935.66	1.5	2804.5	8.44	58,224.1	O49258	12S globulin
5.93 × 10^6^	KGTLDGGINHEGIQYYNDL	703.26	1.8	2107.0	4.54	65,039.4	Q38786	Avenacosidase 1
5.50 × 10^6^	FLVQQCSPVAAVSFLRSQILQQSSCQ	956.79	1.9	2867.5	8.07	24,076.7	L0L845	Gliadin-like avenin
2.58 × 10^6^	NNRGEEFGAFTPKFAQTGSQSYRTRE	993.74	2.3	2978.4	8.59	35,722.3	Q38781	Oat storage protein 12S globulin (Fragm)
Amy								
1.82 × 10^7^	LQQVTQGIFQPQMQGQIEGMRAFA	903.08	3.2	2706.4	6	25,275.1	P80356	Avenin-3
1.61 × 10^7^	MAQLFGQSSTPWQSSRQGG	685.03	1.0	2053.0	9.5	61,861.4	Q38779	11S globulin
1.17 × 10^7^	QQQQQQQPFVQQQQMF	683.35	1.1	2049.0	5.52	24,012.3	L0L6K1	Gliadin-like avenin
9.98 × 10^6^	LQLQQQVFQPQLQQQVFQPQL	855.81	2.8	2566.4	5.52	25,471.1	Q09072	Avenin
8.33 × 10^6^	TFNEPHSFCGLGYGTGLHAPGAR	796.91	2.1	2389.1	6.61	65,039.4	Q38786	Avenacosidase 1
7.93 × 10^5^	YFDEQNEQFRCTG	546.07	3.5	1636.7	4.14	61,861.4	Q38779	11S globulin
Cxl								
1.38 × 10^7^	LQALPAMCDVYVPPHCPVATTPXGF	918.14	2.0	2753.4	5.08	24,076.7	L0L845	Gliadin-like avenin
Ctrl 6.73 × 10^6^	KIQSQNDQRGEIIRV	595.10	1.0	1783.9	8.75	58,674.5	O49258	12S globulin

#: Accession number reported in UniprotKB.

**Table 6:** Contents of phenolic acid (µg/g DW) and AVNs (ppb) in different oat samples (mean ± SD, *n* = 3).

**Polyphenols (*m*/*z*)**	**Oat_Ctrl** **(Mean ± SD)** **(µg/g DW)**	**Oat_Mix** **(Mean ± SD)** **(µg/g DW)**	**Oat_Amy** (**Mean ± SD)** **(µg/g DW)**	**Oat_Cxl** **(Mean ± SD)** **(µg/g DW)**
Cinnamic acid (149.2)	12.1 ± 0.4	4.7 ± 0.7 ^(a)^	5.1 ± 0.4 ^(a)^	6.6 ± 0.2 ^(a)^
p-Coumaric acid (165.2)	63.1 ± 4.1	20.1 ± 8.9 ^(c)^	60.4 ± 0.1 ^ns^	16.4 ± 1.2 ^(a)^
Vanillic acid (169.1)	407.6 ± 60.7	94.3 ± 23.8 ^c)^	228.5 ± 78.1 ^(d)^	178.0 ± 21.7 ^(c)^
Gallic acid (171.1)	2894.3 ± 435.5	806.4 ± 4.5 ^(c)^	1241.1 ± 114.6 ^(c)^	579.7 ± 60.9 ^(b)^
Caffeic acid (181.2)	4.7 ± 0.8	262.2 ± 30.9 ^(b)^	18.5 ± 2.0 ^(b)^	265.3 ± 37.0 ^(b)^
Ferulic acid (195.2)	1.9 ± 0.1	0.7 ± 0.1 ^(b)^	0.7 ± 0.1 ^(b)^	0.99 ± 0.03 ^(b)^
**AVN (*m*/*z*)**	**Oat_Ctrl** **(Mean ± SD)** **(ppb)**	**Oat_Mix ^(b)^** **(Mean ± SD)** **(ppb)**	**Oat_Amy ^(a)^** **(Mean ± SD)** **(ppb)**	**Oat_Cxl ^(c)^** **(Mean ± SD)** **(ppb)**
AVN A (300.1)	6.4 ± 0.8	14.8 ± 2.8 ^(c)^	9.6 ± 0.9 ^(d)^	11.1 ± 4 ^ns^
AVN B (330.1)	45.6 ± 8.8	12.8 ± 1.2 ^(d)^	4.3 ± 0.3 ^(d)^	6.0 ± 0.8 ^(d)^
AVN C (316.1)	7.9 ± 2.2	10.9 ± 3.5 ^ns^	5.6 ± 0.8 ^ns^	26.4 ± 0.5 ^(c)^

The statistical analysis was performed by T-parametric test. ^(a)^ *p* < 0.0001, ^(b)^ *p* < 0.006, ^(c)^ *p* < 0.004, ^(d)^ *p* < 0.05, ns: not significant versus Oat_Ctrl.

## Data Availability

The datasets generated for this study are available on request to the corresponding author.

## Supplementary Materials

The following Supplementary Materials are available online at https://www.mdpi.com/article/10.3390/foods10112695/s1, Table S1: Peptide sequences of 3 kDa identified in oat press cake proteins. Figure S1: (A) TIC of six polyphenols and the AVNs. MS/MS spectra of (B) ferulic acid, (C) gallic acid, (D) p-coumaric acid, (E) caffeic acid, (F) cinnamic acid, (G) vanillic acid, (H) AVN A, (I) AVN B, and (J) AVN C.

## Abbreviations

AA	amino acid
ACN	acetonitrile
AMY	amylase
AVNs	avenanthramides
CD	circular dichroism
DTNB	(5,5-dithio-bis-(2-nitrobenzoic acid)
FA	formic acid
LMW	low molecular weight
MIX	amylase/cellulase/protease
FeIII-TPTZ	ferric tripyridyltriazine
FRAP	ferric reducing ability
HPLC–MS/MS	high-performance liquid chromatography–tandem mass spectrometry
OPA	o-phthalaldehyde
PA	phytic acid
SH	free sulfhydryl group
Tris–HCl	Tris(hydroxymethyl)aminomethane hydrochloride
