# Supplementary material for: Composition of the Protein Ingredients from Insoluble Oat Byproducts Treated with Food-Grade Enzymes, Such as Amylase, Cellulose/Xylanase, and Protease
Source: Foods. 2021 Nov 4;10(11):2695. doi: 10.3390/foods10112695 (PMC8623069; doi:10.3390/foods10112695)

## Slide 1
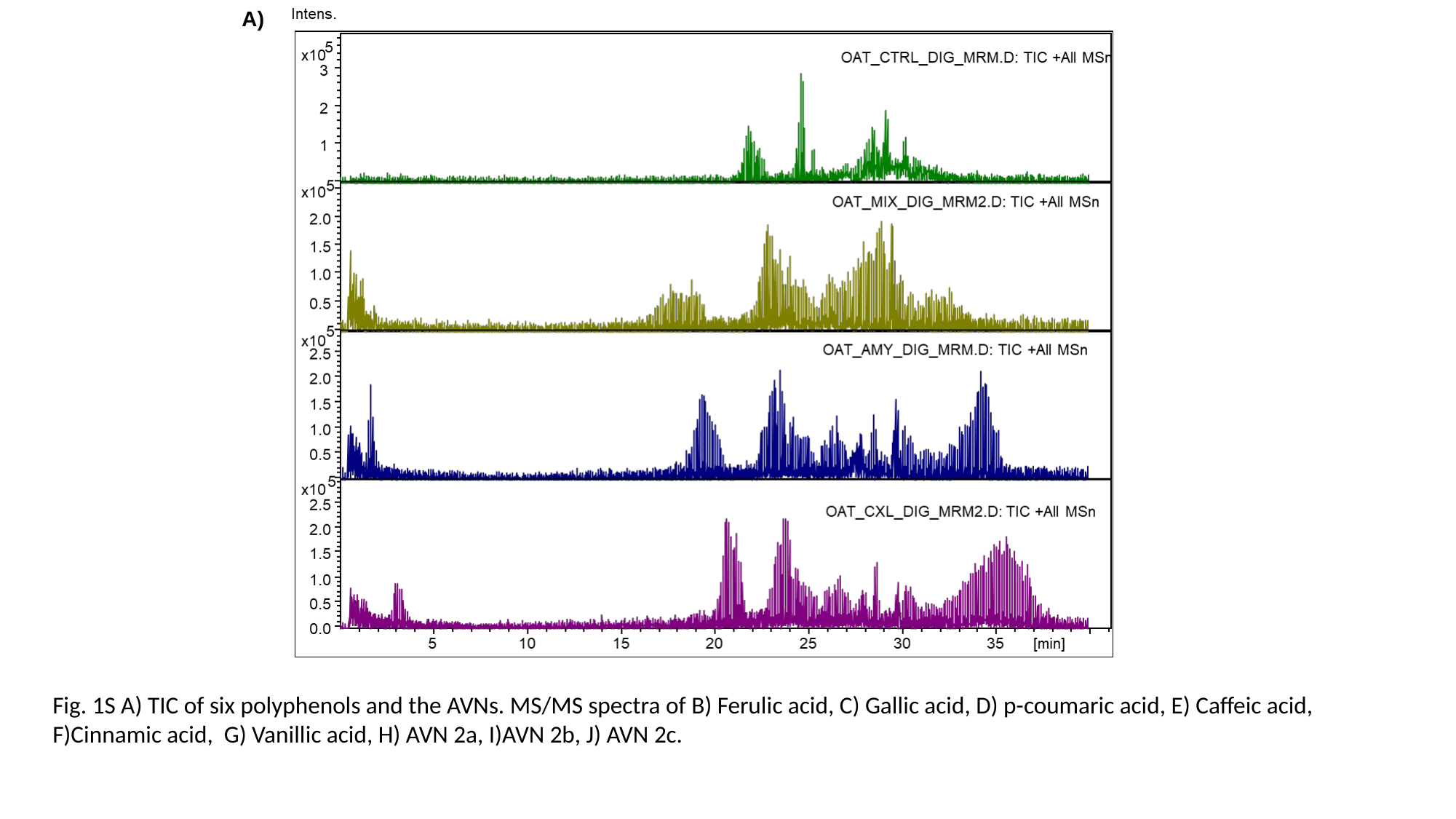

A)
Fig. 1S A) TIC of six polyphenols and the AVNs. MS/MS spectra of B) Ferulic acid, C) Gallic acid, D) p-coumaric acid, E) Caffeic acid, F)Cinnamic acid, G) Vanillic acid, H) AVN 2a, I)AVN 2b, J) AVN 2c.

## Slide 2
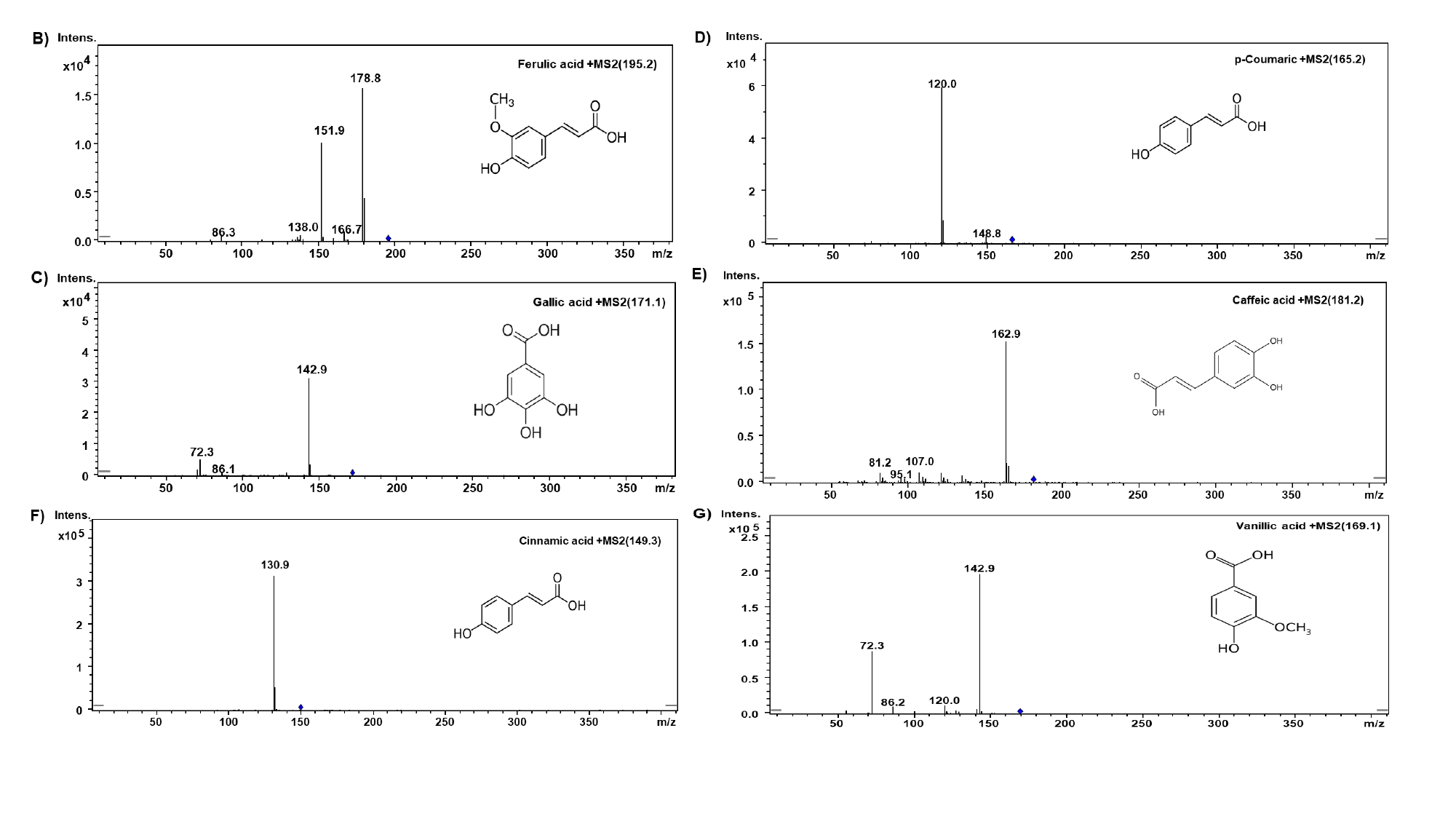

## Slide 3
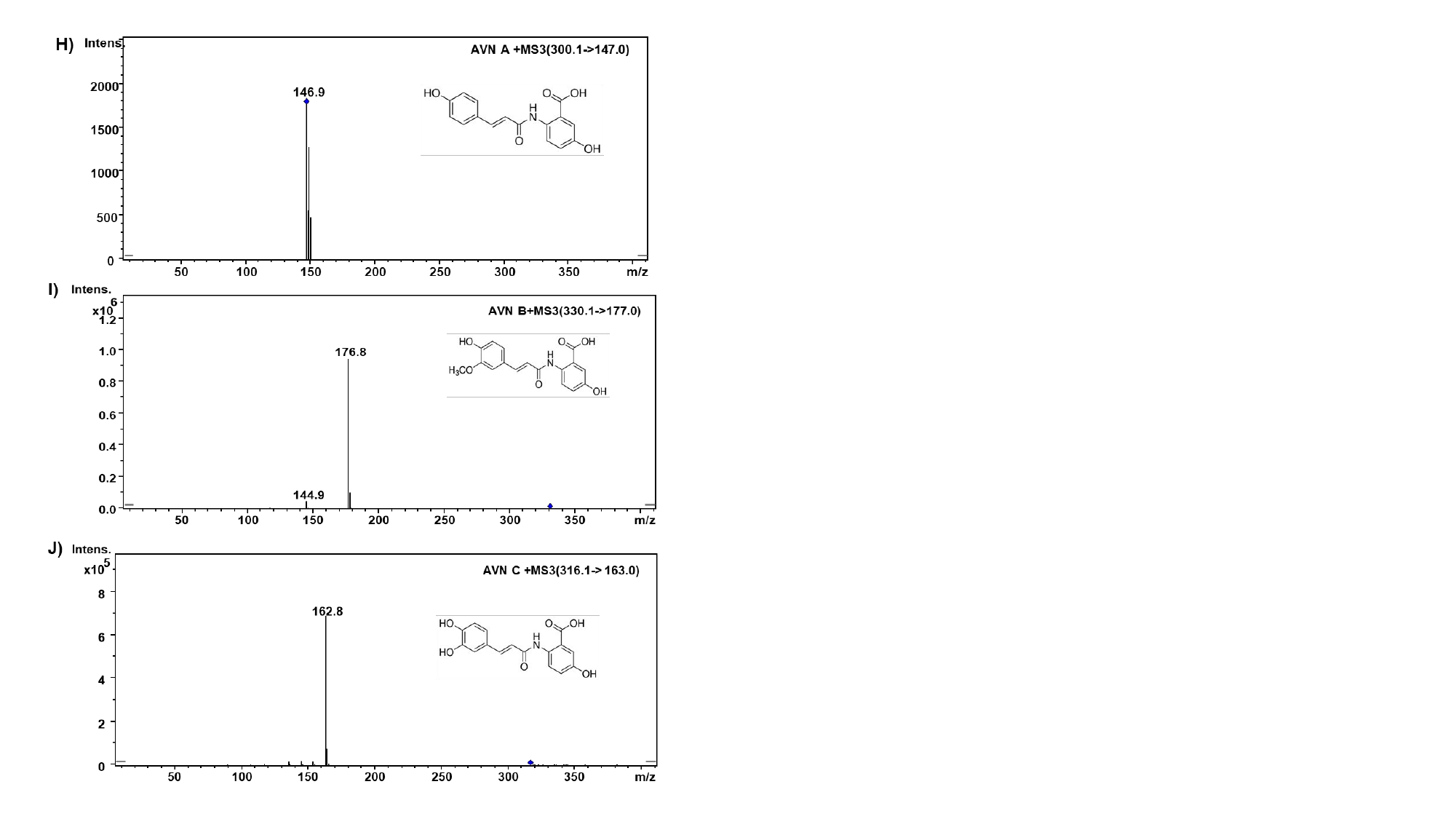

Supplement: Supplementary file 1 [file foods-10-02695-s001.zip › Supplementary/Fig.1S.pptx]
